# Impact of modified short-term fasting and its combination with a fasting supportive diet during chemotherapy on the incidence and severity of chemotherapy-induced toxicities in cancer patients - a controlled cross-over pilot study

**DOI:** 10.1186/s12885-020-07041-7

**Published:** 2020-06-22

**Authors:** Stefanie Zorn, Janine Ehret, Rebecca Schäuble, Beate Rautenberg, Gabriele Ihorst, Hartmut Bertz, Paul Urbain, Anna Raynor

**Affiliations:** 1 Department of Medicine I, Medical Center – University of Freiburg, Faculty of Medicine, University of Freiburg, Freiburg, Germany; 2 Department of Gynecology and Gynecologic Oncology, Medical Center – University of Freiburg, Faculty of Gynecology, University of Freiburg, Freiburg, Germany; 3 Clinical Trials Unit, Medical Center – University of Freiburg, Faculty of Medicine, University of Freiburg, Freiburg, Germany; 4 Department of Hematology, Oncology and Stem Cell Transplantation, Medical Center - University of Freiburg, Faculty of Medicine, University of Freiburg, Freiburg, Germany

**Keywords:** Breast cancer, Gynaecological cancer, Chemotherapy, Fasting, Ketogenic diet, Calorie restriction, Toxicity, Side effects, Pilot study, Insulin-like growth factor

## Abstract

**Background:**

This pilot trial aimed to investigate whether modified short-term fasting (mSTF) reduces the incidence of chemotherapy-induced toxicities and whether an initial ketogenic diet (KD) as fasting supportive diet reduces fasting-related discomfort and improves the compliance.

**Methods:**

In this controlled cross-over trial, gynaecologic cancer patients undergoing chemotherapy with a minimum of 4 cycles fasted for 96 h during half of their chemotherapy cycles and consumed a normocaloric diet during the other chemotherapy cycles. The caloric intake during mSTF was restricted to 25% of each patient’s daily requirement. In addition, half of the patients should eat a 6-day normocaloric KD prior to each mSTF period to investigate a KD’s hunger-suppression effect. Chemotherapy-induced toxicities, fasting-related discomfort, body composition, quality of life, laboratory values, and compliance were assessed at each chemotherapy.

**Results:**

Thirty patients aged 30–74 years (median 54 years) completed the study. During mSTF the frequency and severity score of stomatitis [− 0.16 ± 0.06; 95% CI -0.28 - (− 0.03); *P* = 0.013], headaches [− 1.80 ± 0.55; 95% CI -2.89 – (− 0.71); *P* = 0.002], weakness [− 1.99 ± 0.87; 95% CI -3.72 – (− 0.26); *P* = 0.024] and the total toxicities’ score were significantly reduced [− 10.36 ± 4.44; 95% CI -19.22 - (− 1.50); *P* = 0.023]. We also observed significantly fewer chemotherapy postponements post-mSTF, reflecting improved tolerance of chemotherapy [− 0.80 ± 0.37; 95% CI -1.53 – (− 0.06); *P* = 0.034]. A significant reduction in mean body weight by − 0.79 ± 1.47 kg during mSTF was not compensated and remained until study’s conclusion (*P* < 0.005). On average, Insulin [− 169.4 ± 44.1; 95% CI -257.1 – (− 81.8); *P* < 0.001] and Insulin-like growth factor 1 levels [− 33.3 ± 5.4; 95% CI -44.1 – (− 22.5); *P* < 0.001] dropped significantly during fasting. The KD as a fasting supportive diet neither reduced fasting-related discomfort nor improved compliance of our fasting regimen.

**Conclusion:**

MSTF is safe and feasible in gynaecologic cancer patients. Our results indicate that mSTF during chemotherapy can reduce chemotherapy-induced toxicities and enhance the tolerance of chemotherapy. Larger clinical trials are required to recommend mSTF for cancer patients.

**Trial registration:**

germanctr.de: DRKS00011610, registered 30 January, 2017.

## Background

Periods of intentional fasting are practised worldwide, usually for traditional, cultural, or religious reasons. Fasting is defined as the partial or total cessation of food intake for a specific period. There is ample empirical and observational evidence that medically supervised modified fasting lasting one to 3 weeks is effective in the treatment of several chronic and acute diseases, eg, rheumatism, hypertension, and metabolic syndrome [1]. Recent animal experiments revealed that short-term fasting (STF) prior to high-dose chemotherapy decreases chemotherapy-induced toxicities dramatically without weakening the therapeutic effect [2]. Acute chemotherapy-induced toxicities can trigger to premature discontinuation and dose reductions in chemotherapy, and both are risk factors for poorer therapeutic outcome. Adjuvant metabolic nutritional therapies during cancer treatment such as STF or a ketogenic diet (KD) have recently been discussed and promoted in major newspapers, telecasts, and online, making it a popular topic.

In 2012, Safdie et al. [3] demonstrated that fasting for 48 h sensitised murine, rat, and human glioma cells, but not primary mixed glia cells, to chemotherapy. In the same year, Lee et al. [4] demonstrated that treatment under fasting conditions sensitised 15 of 17 mammalian cancer cell lines to chemotherapeutic agents and was as effective as chemotherapeutic agents in delaying the progression of different tumours. In neuroblastoma mouse models, fasting cycles plus chemotherapy drugs - but not either treatment alone - resulted in long-term cancer-free survival [3]. A recent article by Brandhorst et al. [2] described stress resistance in mice fed either an ad libitum standard diet or macronutrient-defined dietary restriction for 3 days or 60 h fasting prior to high-dose doxorubicin treatment. In contrast to the ad libitum-fed mice, the great majority of fasting (60 h) mice survived to day 25 after chemotherapy (16% vs. 89%) and exhibited no visible signs of stress or pain, like reduced mobility, ruffled hair, and hunched-back posture. Raffaghello et al. [5] showed similar results, namely that fasting for 48–60 h before etoposide treatment enhance resistance in mice.

Fasting induces wide-ranging changes in metabolic pathways and cellular processes, including decreases in circulating insulin-like growth factor-1 (IGF-1) and glucose. This affects different oncogenes including RAS and the AKT-signalling pathway, and leads to downregulation of proliferation and cell growth [6]. Cell culture experiments showed that healthy cells are protected from treatment toxicity, while tumour cells become more vulnerable to chemotherapy during short-term fasting. This phenomenon is described as differential stress resistance. Normal cells enter an alternate state characterised by reduced or a lack of cell division and resistance to multiple stresses, upregulation of DNA repair and induced autophagy. Tumour cells are unable to activate a protective response, and growth pathways remain persistently overactivated. Tumour cells are thus more sensitive to chemotherapy [4–7].

In a case series report published in 2012 [8], patients who fasted voluntarily during chemotherapy proved the feasibility and safety of various fasting regimens and reported significantly fewer chemotherapy-induced side effects, including asthenia, fatigue and gastrointestinal problems such as vomiting and diarrhoea. In the patients whose cancer progression could be followed, there was no evidence that fasting protected tumours or interfered with chemotherapeutic potency. De Groot et al. [9] published the first randomised controlled pilot trial evaluating STF’s feasibility and effects on tolerance of adjuvant chemotherapy in HER2-negative breast cancer patients (N = 13), demonstrating that a 48 h fasting period during chemotherapy was well tolerated and reduced both bone marrow toxicity and chemotherapy-induced DNA damage in peripheral blood mononucleated cells. Bauersfeld et al. [10] found that STF during chemotherapy was safe and well tolerated, and seemed to improve quality of life and fatigue in a pilot trial with 34 gynaecological cancer patients. Dorff et al. [11] published their results of a dose-escalation fasting-study in 2016 where 20 patients with advanced cancer undergoing platinum-based combination chemotherapy fasted for 24, 48 and 72 h during chemotherapy administration, and found that fasting for 72 h was safe and feasible for cancer patients and that fasting-related toxicities were limited to ≤grade 2. Subjects who fasted for 48 h or longer revealed less DNA damage in leukocytes and IGF-1 levels decreased in all fasting cohorts.

Michalsen et al. [12] demonstrated in a non-randomised trial with 209 patients suffering from chronic pain, that STF has no serious side effects.. Typical complaints reported in the initial fasting period at a level that did not interfere with daily activities include hunger, tiredness, irritability, headache, and light-headedness [8, 12, 13]. Fasting-related discomfort occurred especially on fasting days 2 and 3, when metabolism moves to physiological ketosis [13]. Fasting leads to strong neuroendocrine adaptations that resemble the metabolic responses to a KD [14]. A KD involves the intake of a high-fat, adequate-protein and very low- carbohydrate regimen (< 30–40 g/day), and induces a metabolic condition called “physiological ketosis”, namely the state of elevated levels of ketone bodies in the blood [15]. Previous studies revealed that ketosis exerts a hunger-suppression effect, although its mechanisms of action on reducing appetite are not yet completely understood [16–18]. We conducted this pilot study to evaluate the influence of 96 h-fasting on chemotherapy-induced toxicities in patients with gynaecological cancer. Furthermore, quality of life, fatigue, fasting-related discomfort, compliance, nutritional status and laboratory values are assessed. We are the first to have investigated whether a KD as a fasting supportive diet can reduce fasting-related discomfort during the first days and increase patient’scompliance with our fasting regimen.

## Methods

### Study population

We recruited adult women with first diagnosis or first recurrence of histologically confirmed gynaecologic cancers in all stages and undergoing neoadjuvant or adjuvant chemotherapy with a minimum of 4 cycles of the same chemotherapy protocol at a 3- to 4-week interval administrated within 24 h. Our exclusion criteria were: a current state of malnutrition (Nutritional Risk Screening > 3, weight loss > 5% in the last 3 months, body mass index < 18.5 kg/m^2^), eating disorders, diabetes mellitus undergoing drug therapy, gout, severe cardiovascular diseases, pregnancy or lactation, parenteral nutrition, administration of steroids or IGF-1-receptor blockers. Patients were enrolled between March 2017 and December 2017 in the Department of Gynecology and Gynecologic Oncology at the University Medical Center Freiburg by employees of the Department of Nutritional Medicine and Dietetics. All study participants gave their written informed consent. The study protocol was reviewed and approved by the Ethics Committee of Albert-Ludwig University Freiburg (313/16), and the study was registered at germanctr.de as DRKS00011610.

### Study design and intervention

**Fig. 1:**
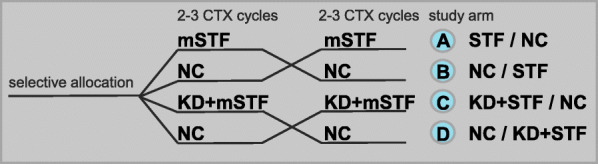
Crossover study design to test the effect of modified short-term fasting (mSTF) during 2 or 3 cycles of chemotherapy (CTX) depending on CTX regimen on CTX-induced toxicities compared to a normocaloric diet (NC) during 2 or 3 cycles of CTX as control and to test the effect of a normocaloric ketogenic diet (KD) followed by mSTF on fasting realated discomfort compared to mSTF alone

This pilot study had an interventional, open-label cross-over design entailing a dietary intervention. Its primary objective was to evaluate the influence of modified short-term fasting (mSTF) on chemotherapy-induced toxicities in gynaecologic cancer patients on the basis of the probability of adverse events grade III and higher. The dietary intervention consisted of an mSTF or a 6-day normocaloric KD prior to each mSTF. Patients were selectively assigned to one of the four study groups: A, B, C, D (Fig. 1). All study groups included 2–3 periods of mSTF or mSTF combined with KD and normocaloric diet (NC), respectively. Patients in study groups A und C started with mSTF or KD prior to mSTF for the first half of chemotherapy cycles, whereas groups B and D began with NC. For statistical analysis we mainly compared NC cycles to mSTF cycles. Furthermore, we compared cycles of mSTF versus cycles of mSTF combined with KD. All patients entered the preparation period after randomisation, which consisted of individually teaching units and the development of an individual diet plan for the 4 day- mSTF period by a skilled nutritional scientist. One day before each dietary intervention period, patients received a reminder by phone or email. In periods between the dietary interventions close to the chemotherapy cycles, patients were on their usual NC.

During mSTF, the level of caloric restriction was limited to 25% of each patient’s daily calorie requirement, consisting of the resting energy expenditure multiplied by a factor that corresponds to the individual overall physical activity level. The resting energy expenditure was estimated with the improved Harris-Benedict equation [19]. The energy content of the mSTF for most patients was between 400 and 600 kcal/day. The mSTF was an individually adapted, very low-calorie diet whose macronutrients revealed to a ketogenic composition (75% fat, 15% protein and 10% carbohydrates) aiming to support the metabolic changes in fasting and to minimise the burden of water-only fasting. The patients were instructed to drink a daily minimum of 2.5 L of any calorie-free liquids, including water, herbal tea, and diet drinks without stimulants such as caffeine and alcohol. The fasting period took place on 5 consecutive days lasting a total of just 4 days (96 h). According to the hypothesis of the differential stress resistance, the greatest protecting effects of fasting are expected when patients are in a state of ketosis. Usually, patients enter the phase of ketosis after 2 or 3 days of fasting. A fasting duration of 96 h was chosen to ensure that the metabolism of all patients has adjusted to fasting and that patients are in the state of ketosis at the time of chemotherapy administration. MSTF started 3 days prior to chemotherapy in the evening at 6 pm. The fasting period ended 1 day after chemotherapy at 6 pm, approximately 24 h after the end of drug administration to extend beyond the half-life of most toxic drugs [20].

While patients in study groups A and B received only a mSTF, patients in study groups C and D additionally obtained a 6-day normocaloric KD prior to each mSTF period. Both fasting and normocaloric KD lead to similar metabolic changes [21] including a state of ketosis which can suppress hunger [16–18]. Accordingly, fasting metabolism was induced via a KD before each 4-day mSTF period. The patients in study groups C and D received advice and recipes to follow a KD ad libitum according to their own food preferences. The patients were encouraged to limit their carbohydrate intake to a maximum of 20–40 g/day and to derive at least 75% of total consumed energy from fat. The macronutrient content during KD was the same as during the mSTF period except for the energy content.

### Outcome assessment

Our outcome measures were determined at baseline, at each chemotherapy cycle and as follow-up 3 weeks after the 4th or 6th cycle depending on the chemotherapy regimen. To determine the main endpoint, we assessed the percentage of patients with grade III or higher chemotherapy-induced toxicities. Toxicities were graded according to the National Cancer Institute Common Terminology Criteria for Adverse Events (NCI CTCAE v4.0) [22]. This comprehensive toxicity grading scale is well established and used in clinical practice. Toxicities were reported by grade (level of severity) on a scale of I to V, whereby grade V (death) is inappropriate for some adverse events. Further outcome measures included chemotherapy-induced toxicities grade I/II (NCI CTCAE v4.0), nutritional status, body composition via bioelectrical impedance analysis (BIA, Body Impedance Analyser Nutriguard-MS™, Data Input GmbH, Germany), and validated questionnaires issued by the European Organisation for Research and Treatment of Cancer (EORTC) and the Functional Assessment of Chronic Illness Therapy (FACIT) focussing on quality of life (EORTC QLQ-C30 [23]), chemotherapy-induced polyneuropathy (EORTC QLQ-CIPN20 [24]) and fatigue (FACIT-Fatigue v4.0 [25]). In addition to the chemotherapy-induced toxicities (NCI CTCAE v4.0), a self-reporting questionnaire was developed especially for this trial to evaluate the chemotherapy-induced side effects in the week following chemotherapy. The patients rated their toxicities daily on a scale of 0 (none) to 3 (severe). Venous blood sample were drawn before each chemotherapy administration and analysed at the Institute for Clinical Chemistry and Laboratory Medicine, University Medical Centre Freiburg. The fasting effect was determined by recording hematologic parameters (differential blood count), inflammatory response [C Reaktive Protein (CRP)], metabolic parameters [insulin, IGF-1] and endocrine parameters [thyroid-stimulating hormone (TSH), free triiodothyronine (fT3), free thyroxine (fT4)].

Our safety measures relied on recommendations published 2013 by the Expert Panel Update of the 2002 Consensus Guidelines for Fasting Therapy [26]; they include laboratory values of electrolytes (sodium, potassium, calcium, magnesium), renal (creatinine, urea nitrogen, uric acid) and liver function (bilirubin, alanine aminotransferase [ALT], aspartate aminotransferase [AST], alkaline phosphatase [ALP], and gamma-glutamyltransferase [GGT]). To document compliance with the mSTF regimen and verify that the control group was not fasting, patients were obliged to record their daily food intake and to monitor their ketosis state by documenting daily measures of urinary ketones via self-testing strips (Ketostix®, Bayer AG, Switzerland) during each fasting cycle. Blood ketone levels were also checked at each chemotherapy via a testing device called FreeStyle Precision Neo Blood Glucose and Ketone Monitoring System with blood β-ketone test strips (Abbott GmbH & Co. KG, Germany). Semi-quantitative food records were analysed by professional software (PRODI®expert v6.5, Nuri-Science GmbH, Germany). At the follow-up visit, a specially developed questionnaire was distributed to evaluate the patients’ subjective feelings and the feasibility of this pilot study.

### Sample size calculation and statistics

As this is a pilot trial, no precise sample size calculation could be made. However, the following considerations regarding its usefulness may justify the choice of 40 patients in a cross-over design with 2 groups (NC vs. mSTF or mSTF + KD) and a binary outcome: probability of adverse events grade III or higher. We assume a probability for grade 3 or higher adverse events of 0.60 (data source: GOIM 9902 study [27]) with NC and of 0.30 with fasting, i.e. a difference in proportions of 0.30. We further assume that the proportion of patients with differing results between NC and mSTF or mSTF + KD would be 0.40 (discordant pairs). A sample size of 30 pairs would have 80% power to detect a difference in proportions of 0.30 when the proportion of discordant pairs is expected to be 0.40 and the method of analysis is a McNemar’s test of equality of paired proportions with a 0.05 two-sided significance level. Taking into account the possibility of dropouts, we would have needed 40 patients to ensure sufficient power.

All variables were tested for normal distribution (Kolmogorow-Smirnow test). Normally distributed variables are presented as mean ± standard deviation and compared using two sample *t*-test for differences between intervention groups or paired *t*-tests for differences between baseline (T_0_) and follow-up (FU). Not normally distributed variables are shown as median (minimum – maximum). The Mann-Whitney test was used for independent groups, and the Wilcoxon rank-sum test for paired groups.

Our primary objective was to evaluate the influence of modified short-term fasting (mSTF) on chemotherapy-induced toxicities based on the probability of adverse events grade III and higher. Unexpectedly, we observed only a very low incidence of CTCAE grade ≥ III during the study period, thus we decided to analyse the probability of toxicities grade I or II as binary variable. Additionally, we analysed the probability of self-reported adverse events as continuous variable on the basis of a point scoring system. Variables measured at several points in time (chemotherapy-induced toxicities, body composition, validated questionnaires, safety and compliance parameters) were analysed as outcomes with a linear mixed model appropriate for the cross-over design, where treatment (NC vs. fasting), period and sequence were treated as fixed effects and patients are considered as random effects. We also adjusted the analysis for group assignment and chemotherapeutic agents. Results are presented as parameter estimates (PE), standard errors (SD) and 95% confidence intervals. The PE represents the difference for each variable between cycles of NC and those of mSTF. Number of chemotherapy cycles were modelled as continuous variable. Statistical significance was set at *P* < 0.05. The data were analysed using IBM SPSS Statistics (Version 24, IBM, New York, USA).

## Results

### Patient characteristics

**Fig. 2:**
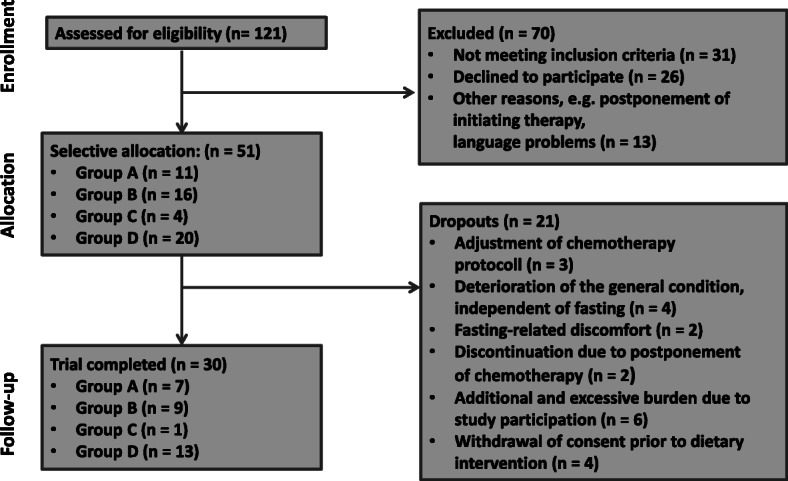
Flow diagram of the study participants from eligibility criteria screening to study completion

From March 2017 until December 2017, 121 women with gynaecological cancers were screened for eligibility, 51 were selectively assigned to one of the four study arms (Fig. 2). Over the study period’s course, 21 patients dropped out for various reasons, including adjustments in their chemotherapy protocol (*n* = 3), deteriorated overall condition independent of fasting (*n* = 4), fasting-related discomfort (*n* = 2), chemotherapy postponements (*n* = 2), additional and excessive burden due to study participation (*n* = 6) or withdrawal of consent before beginning of dietary intervention without any reasons (*n* = 4). Overall, 30 patients completed the intervention study. Seven patients (group A) started with mSTF during the first half of chemotherapy cycles, one patient began with a 6-day normocaloric KD followed by mSTF (group C). Twenty-two patients started with NC during the first half of chemotherapy cycles and changed to one of the fasting interventions afterwards (group B: *n* = 9; group D: *n* = 13). Unexpectedly, patient’s selectively assignment to one of four study groups could not be implemented as originally planned due to the outpatients chemotherapy ward’s strictly scheduled daily routine. Consequently, our groups are of unequal size and the patients’ characteristics differed significantly between groups A-D. Baseline characteristics of the whole study population are summarised in Table 1. The median age of the population was 54 years and ranged from 30 to 74 years. Mean body mass index (BMI) was 26.0 ± 4.6 kg/m^2^. The majority of patients (n = 22, 73,3%) had been diagnosed with breast cancer and undergone a neoadjuvant chemotherapy regimen of Epirubicin/Cyclophosphamid. Further treated tumor entities included endometrial cancer (*n* = 2), ovarian cancer (*n* = 2), and cervical cancer (*n* = 4). According to the nutritional risk screening, 21 patients (70%) had a low risk (NRS = 1), only one patient presented a risk for malnutrition (NRS = 3). In total, patients fasted during 56 of 118 chemotherapy cycles. Daily energy requirement was estimated at 1933 ± 178 kcal per day of NC or KD and 484 ± 44 kcal per fasting days.

**Table 1:** Baseline characteristics of the study cohort

		MW ± SD *n* (%)
Randomisation	Group A	7 (23.3)
	Group B	9 (30.0)
	Group C	1 (3.3)
	Group D	13 (43.3)
Demography	Age (years)	54 ± 11
	Height (m)	1.65 ± 0.07
	BMI (kg/m^2^)	26.0 ± 4.6
Diagnosis	Adjuvant	26 (86.7)
	Metastatic or local advanced	4 (13.3)
Tumour entity	Breast Cancer	22 (73,33)
	Endometrial Cancer	2 (6,7)
	Ovarian Cancer	2 (6.7)
	Cervical Cancer	4 (13,3)
T-categories	T1	10 (33.3)
	T2	14 (46.7)
	T3	3 (10.0)
	T4	3 (10.0)
Nodal status	N0	20 (66.7)
	N1	8 (26.7)
	N2	1 (3.3)
	N3	1 (3.3)
Metastasis	M0	27 (90.0)
	M1	3 (10.0)
Chemotherapy regimens	Paclitaxel/Carboplatin	7 (23.3)
	Epirubicin/Cyclophosphamid	22 (73.3)
	Docetaxel/Cyclophosphamid	1 (3.3)
Treatment	Adjuvant chemotherapy	6 (20)
	Neoadjuvant chemotherapy	22 (73.3)
	Palliative chemotherapy	3 (10.0)
Nutritional status (NRS 2002)	1	21 (70.0)
	2	8 (26.7)
	3	1 (3.3)
Energy requirement	Basal metabolic rate (kcal)	1391 ± 125
	Physical Activity Level	1.39 ± 0.06
	Active metabolic rate (kcal)	1933 ± 178
	25% of the active metabolic rate (kcal)	484 ± 44

### Compliance and diet compositions

Physiological blood ketosis (blood ketone level ≥ 0.6 mmol/l) was detected in 71.4% (n = 40) of the 56 chemotherapy cycles. The ketosis state differed significantly between mSTF and NC (mSTF 1.27 ± 1.18 mmol/l, NC 0.21 ± 1.98 mmol/l; *P* < 0.001). In addition to blood ketosis on the day of chemotherapy administration, patients measured urinary ketosis for daily self-testing during periods of fasting interventions. Mean urinary ketosis on fasting days confirmed the metabolic shift to ketosis and fat oxidation (1.47 ± 1.06 mmol/l). Neither the frequency nor intensity of blood ketosis on fasting days differed significantly among patients in study groups C and D on a 6-day normocaloric KD prior to mSTF compared to patients in study groups A and B with only mSTF.

Mean daily caloric intake was 1631 ± 566 kcal during NC. There were no significant differences in daily energy intake between NC and KD before mSTF. However, macronutrient composition of the KD changed significantly in comparison to NC (*P* < 0.001) with higher fat (KD 64%; NC 39%) and protein (KD 20%; NC 17%) as well as lower carbohydrate consumption (KD 16%; NC 44%), respectively. Mean daily caloric intake on fasting days was 493 ± 157 kcal and in line with the estimated energy requirements. While mSTF, the intake of macronutrient composition resembled the KD’s with 58% energy intake from fat, 23% from carbohydrates and 19% from proteins. We observed no significant differences in macronutrient composition on fasting days between study arms C and D (KD prior to mSTF) and study arms A and B (mSTF only).

### Toxicity

We found that patients experienced significantly less frequent grade I/II stomatitis during mSTF cycles compared to NC cycles [− 0.16 ± 0.06; 95% CI -0.28 - (− 0.03); *P* = 0.013, Table 2]. Regarding our primary endpoint, namely the percentage of patients with grade III or higher chemotherapy-induced toxicities, we documented only one case of CTCAE grade III nausea during an mSTF cycle. Our study therefore failed to meet its primary endpoint. The most frequent toxicities grade I/II were fatigue (41.6%), nausea (33.6%) and stomatitis (15.9%).

**Table 2:** Chemotherapy related toxicities according to CTCAE (Grade I/II) comparing cycles of mSTF (n = 56) with cycles of NC (*n* = 62)

	NC	mSTF	PE ± SD*	95% CI	*P-*Value
Infection	2 (3.4%)	4 (7.3%)	0.07 ± 0.04	0.01–0.15	0.097
Fatigue	22 (37.9%)	25 (45.5%)	-0.01 ± 0.09	− 0.20 – 0.17	0.882
Insomnia	2 (3.4%)	4 (7.3%)	0.03 ± 0.04	− 0.05 – 0.11	0.505
Headaches^a^	2 (5.7%)	0	−0.03 ± 0.06	− 0.14 – 0.09	0.649
Dizziness^a^	3 (8.6%)	2 (7.1%)	−0.10 ± 0.07	−0.25 – 0.05	0.187
Depression	2 (3.4%)	2 (3.6%)	− 0.02 ± 0.04	− 0.10 – 0.06	0.560
Nausea	22 (37.9%)	16 (29.1%)	− 0.01 ± 0.10	−0.21 – 0.18	0.895
Vomiting	0	2 (3.6%)	0.02 ± 0.03	−0.03 – 0.08	0.413
Diarrhea	2 (3.4%)	2 (3.6%)	−0.01 ± 0.04	−0.09 – 0.07	0.877
Obstipation^b^	5 (8.8%)	4 (7.3%)	−0.02 ± 0.06	−0.13 – 0.09	0.688
Stomach pains^a^	1 (2.9%)	0	−0.02 ± 0.04	−0.10 – 0.07	0.695
Reduced appetite^a^	7 (20.0%)	2 (7.1%)	−0.16 ± 0.11	−0.39 – 0.06	0.150
Hunger^c^	2 (5.6%)	0	−0.03 ± 0.06	−0.15 – 0.09	0.606
Stomatitis	15 (25.9%)	3 (5.5%)	−0.16 ± 0.06	−0.28 – (− 0.03)	0.013
Esophagitis	2 (3.4%)	2 (3.6%)	0.01 ± 0.04	− 0.08 – 0.09	0.892
Neuroses	8 (13.8%)	10 (18.2%)	0.07 ± 0.07	−0.06 – 0.20	0.280
Arthralgia	1 (1.7%)	2 (3.6%)	0.02 ± 0.03	−0.05 – 0.09	0.614
Pain	11 (19%)	3 (5.5%)	− 0.11 ± 0.06	−0.23 – 0.01	0.075
Dyspnea	0	2 (3.6%)	0.03 ± 0.02	− 0.01 – 0.07	0.098
Oedemas	0	1 (1.8%)	0.0 ± 0.02	−0.04 – 0.04	0.840

Values are shown as mean ± SD. Due to missing data, we included data of 58 cycles of NC and 55 cycles of mSTF in this analysis.*PE ± SD represents the difference for CTCAE points between cycles of NC and those of mSTF. All side effects were scored according to CTCAE v.4.0. Each side effect was scored once per patient during each chemotherapy cycle. a *n* = 63; b n = 112; c *n* = 64

In addition to the incidence of CTCAE-documented side effects by physicians, patients reported their chemotherapy-induced side effects in the week following chemotherapy (Table 3). Besides thelower frequency and severity scores of self-reported headaches [− 1.80 ± 0.55; 95% CI -2.89 – (− 0.71); *P* = 0.002], the frequency and severity score of self-reported feeling weak decreased significantly during mSTF cycles compared to those of NC [− 1.99 ± 0.87; 95% CI -3.72 – (− 0.26); *P* = 0.024]. Furthermore, the frequency and severity score of total self-reported toxicities were significantly reduced during mSTF cycles compared to NC cycles [− 10.36 ± 4.44; 95% CI -19.22 - (− 1.50); P = 0.023]. We detected no significant differences in the incidence and severity of adverse events between mSTF alone or in combination with a prior KD in either the documented CTCAE or self-reported chemotherapy-induced toxicities.

**Table 3:** Self-reported chemotherapy-induced toxicities 1 week after chemotherapy comparing cycles of mSTF (*n* = 56) with cycles of NC (*n* = 62)

	NC	mSTF	PE ± SD*	95% CI	*P-*Value
Reduced Appetite	4.51 ± 5.01	2.75 ± 4.75	− 1.01 ± 0.71	−2.43 – 0.42	0.164
Hunger	5.36 ± 5.48	4.98 ± 5.23	− 0.14 ± 0.60	−1.35 – 1.06	0.813
Nausea	3.72 ± 4.34	4.24 ± 4.91	− 0.13 ± 0.57	−1.26 – 0.99	0.814
Vomiting	0.21 ± 0.84	0.45 ± 1.14	0.32 ± 0.19	− 0.07 – 0.70	0.106
Stomach pains	1.66 ± 3.25	2.25 ± 3.69	0.79 ± 0.63	− 0.47 – 2.04	0.215
Diarrhea	1.00 ± 2.88	0.78 ± 2.34	− 0.41 ± 0.58	−1.57 – 0.76	0.489
Obstipation	3.17 ± 4.42	3.06 ± 3.83	− 0.73 ± 0.84	− 2.41 – 0.94	0.385
Fever	0.11 ± 0.51	0.14 ± 0.98	0.13 ± 0.18	− 0.23 – 0.48	0.480
Headaches	2.74 ± 4.30	1.18 ± 2.06	− 1.80 ± 0.55	−2.89 – (− 0.71)	0.002
Insomnia	5.21 ± 5.35	4.18 ± 4.97	− 1.18 ± 0.77	−2.73 – 0.36	0.130
Fatigue	7.96 ± 5.52	6.63 ± 5.66	− 1.45 ± 0.85	−3.15 – 0.26	0.094
Dizziness	2.23 ± 9.19	2.84 ± 4.15	− 0.13 ± 0.59	−1.31 – 1.05	0.832
Weakness	7.11 ± 5.40	5.78 ± 5.26	−1.99 ± 0.87	−3.72 – (− 0.26)	0.024
Exhaustion	6.74 ± 5.46	6.20 ± 5.67	−1.23 ± 0.85	− 2.92 – 0.46	0.152
Total toxicities	56.36 ± 32,14	47.52 ± 33.21	−10.36 ± 4.44	−19.22 – (− 1.50)	0.023

Values are shown as mean ± SD. Due to missing data, we included data of 53 cycles of NC and 51 cycles of mSTF in this analysis. * PE ± SD represents the difference for each toxicity score points between cycles of NC and those of mSTF. Per day, patients awarded each toxicity with 0 (none) to 3 (severe) points. Consequently, each toxicity received 0–21 points. The scores of all toxicities in the following 7 days after chemotherapy are summarised in total toxicities (0–294 points)

Despite the fact that mSTF was safe, patients reported low grade fasting-related side effects. Overall, the most common reported fasting-related side effects included hunger (*n* = 8), dizziness (*n* = 5), weakness (*n* = 4), and headaches (*n* = 4).

Unfortunately, chemotherapy-induced toxicities frequently cause patients to postpone chemotherapy, an additional and extremely burdensome stress factor for patients. We compared the number of days of postponements during mSTF cycles with those of NC. Patients experienced significantly fewer chemotherapy postponements during mSTF cycles [− 0.80 ± 0.37; 95% CI -1.53 – (− 0.06); *P* = 0.034], reflecting better chemotherapy tolerance.

### Weight and body composition

Comparing cycles of mSTF to NC cycles, we observed a significant loss in mean BIA fat mass [− 0.63 ± 0.23; 95% CI -1.09 – (− 0.17); *P* = 0.008], leading to significant weight loss during mSTF [− 0.84 ± 0.26; 95% CI -1.35 – (− 0.33); *P* = 0.002]. Apart from BIA fat mass, body composition remained on average constant. In contrast to our baseline measures, lost body weight and fat mass during mSTF was not counterbalanced, and remained significantly reduced at the end of the study (*P* < 0.005). Mean body weight and mean fat mass were 71.4 ± 12.3 kg and 23.0 ± 8.8 kg at the beginning and 69.8 ± 11.6 kg and 21.4 ± 8.4 kg at the end of the intervention, respectively. When comparing body composition in our total study population at baseline with that at follow-up, we noted that mean BIA body cell mass and mean BIA phase angle demonstrated a significant reduction from 23.2 ± 3.2 kg to 22.1 ± 3.0 kg (*P* = 0.007) and from 5.27 ± 0.74 kg to 4.8 ± 0.67 kg (*P* = 0.001), whereas the mean BIA extracellular cell mass revealed a significant increase from 25.2 ± 3.3 kg to 26.8 ± 3.2 kg (*P* = 0.001), respectively. However, there were no significant differences in the anthropometric parameters measured comparing mSTF alone or in combination with a prior KD.

### Blood parameters

All haematological, inflammatory, metabolic and endocrine parameters were compared between mSTF cycles and those of NC (Table 4). Significant differences in haematological parameters were detected in mean corpuscular cell volume [MCV; − 1.68 ± 0.33; 95% CI -2.33 –(− 1.02); *P* < 0.001] and mean corpuscular haemoglobin [MCH; − 0.38 ± 0.13; 95% CI -0.63 –(− 0.13); *P* = 0.004]. Both parameters fell significantly during mSTF cycles compared to NC cycles (Table 4). However, erythrocytes were slightly increased on average during mSTF [0.11 ± 0.06; 95% CI − 0.01 – 0.23; *P* = 0.071]. Regarding leucocytes, thrombocytes and neutrophils, we observed no significant differences in counts between mSTF and NC cycles.

**Table 4:** Blood parameters of chemotherapy cycles with short-term fasting or normocaloric diet comparing cycles of mSTF (*n* = 56) with cycles of NC (*n* = 62)

	Unit	NC	mSTF	PE ± SD	95% CI	*P*-Value
Blood count						
Leukocytes	10^3^/μl	6.67 ± 2.23	6.09 ± 2.25	− 0.25 ± 0.30	−0.84 – 0.35	0.406
Thromobytes	10^3^/μl	288.2 ± 76.6	306.6 ± 76.9	4.85 ± 10.76	−16.56 – 26.25	0.654
Erythrocytes	10^3^/μl	3.91 ± 0.47	3.95 ± 0.37	0.11 ± 0.06	−0.01 – 0.23	0.071
Haemoglobin	g/dl	11.7 ± 1.4	11.6 ± 1.1	0.18 ± 0.18	−0.19 – 0.54	0.337
Haematocrit	%	33.5 ± 3.9	33.1 ± 2.9	0.31 ± 0.52	−0.72 – 1.33	0.553
MCH	pg	29.9 ± 1.5	29.5 ± 1.6	−0.38 ± 0.13	−0.63 – (− 0.13)	0.004
MCV	10^− 9^ μl	85.7 ± 4.0	84.1 ± 3.7	−1.68 ± 0.33	−2.33 – (− 1.02)	< 0.001
MCHC	g/dl	34.9 ± 0.9	35.1 ± 0.9	0.23 ± 0.14	− 0.05 – 0.51	0.109
Neutrophils	10^3^/μl	4.70 ± 2.20	4.29 ± 2.24	−0.17 ± 0.30	−0.76 – 0.43	0.581
Lymphocytes	%	21.0 ± 10.7	19.7 ± 12.4	−0.89 ± 1.32	− 3.50 – 1.73	0.503
Monocytes	%	8.22 ± 4.70	10.1 ± 5.3	0.90 ± 0.72	−0.52 – 2.33	0.210
Eosinophils	%	0.71 ± 1.29	0.51 ± 1.18	−0.05 ± 0.19	−0.42 – 0.33	0.813
Basophils	%	0.68 ± 0.76	0.74 ± 0.59	−0.02 ± 0.10	−0.18 – 0.22	0.860
Electrolytes						
Potassium	mmol/l	4.29 ± 0.42	4.28 ± 0.32	−0.01 ± 0.07	−0.15 – 0.12	0.845
Sodium	mmol/l	139.2 ± 2.2	137.9 ± 3.3	− 1.17 ± 0.42	− 2.0 – (− 0.33)	0.007
Calcium	mmol/l	2.33 ± 0.14	2.34 ± 0.15	0.02 ± 0.03	−0.04 – 0.07	0.561
Magnesium	mmol/l	0.80 ± 0.07	0.78 ± 0.07	−0.0 ± 0.01	−0.02 – 0.02	0.840
Liver and kidney parameters						
Urea	mg/dl	26.9 ± 10.0	25.3 ± 10.6	− 1.087 – 1.34	−4.54 – 0.79	0.165
Creatinine	mg/dl	0.71 ± 0.16	0.72 ± 0.19	0.02 ± 0.01	−0.01 – 0.05	0.171
Uric acid	mg/dl	4.44 ± 1.33	5.98 ± 1.79	1.35 ± 0.17	1.01–1.68	< 0.001
Bilirubin	mg/dl	0.33 ± 0.25	0.29 ± 0.11	0.01 ± 0.03	− 0.05 – 0.07	0.305
GOT	U/l	24.2 ± 8.7	25.0 ± 7.2	1.38 ± 1.48	− 1.56 – 4.32	0.353
GPT	U/l	22.4 ± 11.8	23.5 ± 12.2	−0.10 ± 1.46	−3.01 – 2.81	0.947
AP	g/dl	71.4 ± 22.0	73.6 ± 25.0	− 1.96 ± 2.29	−6.52 – 2.58	0.392
Albumin	g/dl	4.31 ± 0.35	4.29 ± 0.32	0.02 ± 0.06	− 0.09 – 0.13	0.741
CRP	mg/l	5.57 ± 4.93	8.1 ± 13.5	0.87 ± 1.95	− 3.0 – 4.73	0.657
Hormones						
TSH	μU/ml	1.05 ± 0.74	1.03 ± 0.73	−0.1 ± 0.07	−0.24 – 0.05	0.188
fT3	pmol/l	4.21 ± 0.81	3.75 ± 0.76	−0.47 ± 0.09	−0.64 – (− 0.30)	< 0.001
Ft4	pmol/l	15.5 ± 2.7	16.4 ± 2.9	0.82 ± 0.37	0.09–1.55	0.028
Insulin	pmol/l	252.7 ± 372.8	140.1 ± 252.4	− 169.4 ± 44.1	− 257.1 – (− 81.8)	< 0.001
IGF-1	ng/ml	127.7 ± 55.7	97.8 ± 46.4	− 33.3 ± 5.4	−44.1 – (− 22.5)	< 0.001

Values are shown as mean ± SD. Due to missing data, we included data of 61 cycles of NC and 56 cycles of mSTF in this analysis. Abbreviations: *AP* alkaline phosphatase, *CRP* C-reactive protein, *GOT* glutamic-oxaloacetic transaminase, *GPT* glutamic-pyruvic transaminase, *IGF-1* insulin-like growth factor 1, *MCH* mean corpuscular haemoglobin, *MCHC* mean corpuscular haemoglobin concentration, *MCV* mean corpuscular volume, *fT3* free triiodothyronine, *fT4* free thyroxine, *TSH* thyroid stimulating hormone

Although the mean level of sodium in blood was significantly lower during mSTF cycles than during NC cycles [− 1.17 ± 0.42; 95% CI -2.0 –(− 0.33); *P* = 0.007], mean sodium levels remained within the reference range. All other electrolytes remained unchanged throughout the intervention. Monitoring kidney function, we identified significantly elevated mean uric acid levels, exceeding the reference value, during mSTF [1.35 ± 0.17; 95% CI 1.01–1.68; *P* < 0.001]. No significant changes were observed in urea and creatinine values, or in liver parameters and CRP comparing mSTF cycles and NC cycles.

Mean thyroid levels fT3 and fT4 revealed significant changes within the reference range during mSTF cycles compared to those of NC, whereas mean TSH remained unaffected. In contrast to the significantly reduced mean fT3 levels [− 0.47 ± 0.09; 95% CI -0.64 – (− 0.30); *P* < 0.001], mean fT4 rose significantly during mSTF cycles compared to NC cycles [0.82 ± 0.37; 95% CI 0.09–1.55; *P* = 0.028]. Both mean Insulin [− 169.4 ± 44.1; 95% CI -257.1 – (− 81.8); *P* < 0.001] and mean the IGF-1 level [− 33.3 ± 5.4; 95% CI -44.1 – (− 22.5); *P* < 0.001] dropped significantly during mSTF cycles compared to NC cycles. By the patients’ follow-up visit, all blood parameters that had altered due to fasting had returned to normal levels. We observed no significant differences in any blood parameter when comparing mSTF alone with mSTF in combination with a prior KD.

### QoL, CIPN and fatigue questionnaires

No significant changes in QoL, CIPN and fatigue were revealed after comparing either mSTF cycles to those of NC or mSTF alone to mSTF in combination with a prior KD. The severity of fatigue remained unchanged at follow-up compared to baseline.

Fasting was generally well tolerated by our patients. At follow-up, 23 patients rated mSTF’s effect on their general health as “very good” or “good”. Only one patient declared mSTF to be “difficult”, while 11 rated it as “quite difficult”. MSTF was evaluated as “quite easy” or “easy” by 9 patients, respectively. In total, more than half the patients would fast again during chemotherapy (*n* = 24).

## Discussion

The primary objective of this clinical trial was to evaluate the effects of a 4-day mSTF with a ketogenic macronutrient composition on chemotherapy-induced toxicities CTCAE grade III and higher in patients with gynaecological cancers. Secondary objectives were the assessment of toxicities CTCAE I/II, self-reported toxicities, body composition, blood parameters and subjective well-being (QoL, CIPN and fatigue). This is the first clinical trial to explore the effects of a 6-day normocaloric KD prior mSTF as a fasting supportive diet on fasting-related discomfort and compliance.

We were surprised to have observed only a very low incidence of CTCAE grade ≥ III during the study period, which meant we were unable to assess our primary endpoint. We did, however, find that mSTF improved patient’s tolerability of chemotherapy thanks to fewer chemotherapy-induced toxicities such as less stomatitis, fewer headaches, less weakness and overall toxicities. In agreement with previous studies, these outcomes confirm that mSTF during chemotherapy is safe and well tolerated [8, 11]. Our findings differed between CTCAE and self-reported toxicities, which may be attributable to the different time points of the queries. Coolbrandt et al. [28] demonstrated that delayed self-assessments of chemotherapy-induced toxicities from retrospective queries as in the CTCAE, provide a distorted, less accurate overview of patient’s actual symptoms and experienced symptom severity than immediate self-reports. Hence, we assume that prospective self-reported toxicities reflect the genuine side effects better than the retrospective CTCAE. Considering previous human fasting studies during chemotherapy, note that there are major differences among study populations, designs, types, and durations of fasting interventions. It is thus not surprising that findings on chemotherapy-induced toxicities vary from trial to trial. While Safdie et al. [8] and Dorff et al. [11] detected fewer gastrointestinal toxicities, including less nausea, vomiting and diarrhea after STF, we observed no differences in these symptoms. This can probably be explained by the fact that our patients tended to report very few cases of gastrointestinal toxicity during both mSTF and NC cycles, due to each patient’s individually-adapted preventive concomitant medication against nausea and vomiting. Safdie et al. [8] and Bauersfeld et al. [10] reported significant reduced fatigue after STF.. However, in line with Safdie et al. [8], our trial patients reported significant less weakness in the week after mSTF, indicating that mSTF could promote regeneration after chemotherapy. Unexpectedly, patients experienced significant fewer headaches during mSTF, even though fasting and irregular meals are among the frequent triggers for migraine [29]. However, there is some evidence that ketones reduce the frequency and severity of migraine and headaches presumably via improved mitochondrial function and less oxidative stress [30–32]. It is well known that many serious chemotherapy-induced side effects compromise the continuation of therapy, leading to its postponement or interruption. Overall, the reduced toxicities due to mSTF in this trial probably resulted in faster physical regeneration after chemotherapy and demonstrably fewer chemotherapy postponements.

Animal studies investigating STF during chemotherapy have demonstrated that STF leads to less damages and faster regeneration in bone marrow cells and resulting cells, especially leukocytes [33]. Safdie et al. [8] confirmed these observations in their first case series report, indicating that especially leukocytes, thrombocytes and neutrophils reveal faster regeneration due to STF during chemotherapy. De Groot et al. [9] observed significantly increased erythrocytes and thrombocytes, and Dorff et al. [11] detected less neutropenia and thrombocytopenia,concluding that STF protects against depletion and DNA damage to leukocytes. Contrary to previous studies, we identified no significant changes in any of the above-mentioned blood parameters, which is probably attributable to our small study population. However, we observed significant reductions in MCH and MCV during mSTF. These blood parameters are considered to be independent prediction parameters of survival in various cancers prior to surgery [34]. In case of breast cancer patients, higher MCH levels suggest adverse effects on disease-free survival [35]. Although reduced MCH and MCV values can be considered positive results, the clinical relevance of these findings remain unclear, particularly in view of this pilot trial’s small patient cohort.

We measured significantly increased ketone body concentrations during mSTF, which proves that patients were fasting-compliant. This finding is consistent with the result of Dorff et al. [11]. While both de Groot et al. [9] and Dorff et al. [11] observed only a slight trend towardlower insulin levels during STF cycles, we detected a significant insulin reduction. Similarly to de Groot et al. [9], we detected a significant reduction in IGF-1 during mSTF. This reduced IGF-1 seems to be one of the key mediators of differential stress resistance in response to nutrient restriction [36]. Previous experimental data indicate that it is mainly low IGF-1 levels that induce differential stress resistance, leading healthy cells to be protected from chemotherapy’s toxicity, and mediated by the downregulation of cell growth and proliferation, while tumour cells are unable to activate a protective response [4, 5, 37]. Since we observed both reductions in IGF-1 and chemotherapy-induced toxicities, our results support the hypothesis that reducing IGF-1 promotes differential stress resistance. Blood glucose levels should be measured in future trials to obtain deeperunderstanding how ketones, insulin, and IGF-1 fluctuate. The increase in endocrine parameter fT4we observed is in line with the results of the human-fasting intervention trial by de Groot et al. [9], and our earlier clinical trial that, investigated the effect of a normocaloric six-week KD on biochemical parameters in healthy adults [38]. While de Groot et al. [9] revealed no change in fT3, we identified a significant reduction in fT3 during KD in the aforementioned trial in healthy subjects [38]. We observed comparable changes in thyroid hormones in a clinical trial with a normocaloric KD and in this trial with mSTF. This can probably be attributed to the state of physiological ketosis, which appears in both mSTF and KD due to carbohydrate restriction. The change in the fasting safety parameters sodium and uric acid during mSTF has been demonstrated in fasting studies with healthy adults [13, 39]. Fasting is known to provoke dehydrating effects, including the enhanced excretion of minerals, especially sodium. Enhanced natriuresis during initial fasting days is accompanied by the excretion of ketones via urine, which is always accompanied by cations, namely initially by sodium, followed by ammonium [40, 41]. The increased uric-acid concentration during fasting is due to reduced renal uric acid clearance via impaired tubular uric acid secretion because ketone bodies compete with uric acid for shared tubular secretion [41, 42].

Consistent with previous fasting studies in healthy adults, we observed a mild weight loss (< 5%) during mSTF, which remained until the end of our examination [13, 39]. In contrast, previous animal and human trials investigating fasting during chemotherapy showed that animals and humans regained their usual weight after an initial weight loss [3–5, 8, 10, 11, 43]. In this respect, note in particular that the documented weight loss in our trial is primarily attributable to lost fat mass, whereas lean body mass remained stable. Compared to water-only fasting, mSTF allowed a slight caloric intake per each fasting day to minimise protein catabolism, including the loss of lean body mass [44]. Given that lost lean body mass is associated with impaired nutritional status and poor general condition, it should be avoided by all means [45, 46]. Although weight loss proved ultimately harmless in our trial, fasting accompanying chemotherapy may only be implemented if the risk of malnutrition can be excluded.

Indeed, this is the first human fasting intervention study to have assessed body composition during fasting via BIA measurements. We thereby observed significant reductions in body cell mass and phase angle over the trial’s course. Generally speaking, phase angle is an independent prognostic factor for impaired functional and nutritional status and overall survival [47, 48]. Reductions in the phase angle during cancer treatment can frequently be explained by chemotherapy-induced damages, because cytostatic drugs reduce total cell amount and membrane integrity [47, 49]. Notably, phase angles were already below reference values for healthy women stratified by age and BMI at the beginning of our study [50]. An adjusted reference value (more precisely, the fifth percentile of the phase angle reference value) is commonly used especially for oncological patients [48, 51]. Nevertheless, the phase angle was slightly below the adjusted reference value at the end of our trial. No published clinical trial to date has investigated fasting’s impact on the phase angle. Moreover, it remains unclear whether fasting affects body composition,, especially the phase angle.

Similarly to Bauersfeld et al. [10], we collected data on QoL, fatigue and additionally CIPN. While Bauersfeld et al. [10] observed significant improvements in fatigue and QoL due to STF during chemotherapy, our results fail to confirm their findings. However, note, that the informative value of the three afore mentioned questionnaires is limited due to an unfavourable point in time for collecting data on the day of chemotherapy, reflecting QoL, CIPN and fatigue in the week before chemotherapy. No valid conclusions can be drawn from our results concerning the impact of fasting on QoL, CIPN, and fatigue in the week following chemotherapy.

Since both mSTF and KD trigger similar metabolic changes, including a state of ketosis that can suppress hunger [16–18], we hypothesized that a KD as a fasting supportive diet prior to mSTF would improve compliance with the fasting regimen and reduce initial fasting-related discomfort. However, we detected no beneficial effects in conjunction with a 6-day normocaloric KD prior to mSTF compared to mSTF alone by relying on compliance, fasting-related discomfort, chemotherapy-induced toxicities and other outcome parameters in this trial. On the contrary, patients consuming a KD prior to mSTF found the feasibility of this intervention to be harder than did patients consuming an mSTF alone. Therefore, a KD prior to mSTF seemed to be an additional burden to patients undergoing chemotherapy, providing neither benefit nor advantage.

Our study had some limitations. The most obvious is our small sample size within the scope of the pilot trial, which limits our study’s power and precludes firm statistical conclusions. The high dropout rate in our pilot trial limits the power of the conclusion. Several patients dropped out because of an additional and excessive burden of the study intervention or without giving reasons before the beginning of the intervention. Not all cancer patients are sufficiently motivated and resilient to rise to the challenge of fasting during chemotherapy. Thus, further fasting studies have to pay great attention to the recruitment and better informing of study participants. Although cross-over models are restricted by carry-over effects, we chose this design for our pilot trial because of the low influence of confounding variables in control and intervention groups and the high statistical efficiency associated with small sample sizes. In our pilot trial, carry-over effects can be neglected because with the end of fasting and return to normocaloric diet the metabolism changes very quickly from hunger metabolism to food intake metabolism. Therefore, washout period of 3 weeks is sufficient. Another limitation is due to missing randomisation. The randomisation was initially planned but due to the outpatient chemotherapy ward’s strictly-set daily routine, it was impossible for us to conduct the randomisation as planned. Consequently, patients were not randomly but selectively assigned to our four study groups. Additionally, we failed to document concomitant medication during chemotherapy in our pilot trial. Finally, it is possible that patients subjectively overestimated fasting’s positive effects due to their knowledge of the allocated interventions and the generally positive attitude toward fasting cures in Germany. This limitation can only be resolved by a double-blinded randomised study design, which is not possible with natural nutrition.

## Conclusion

In summary, our results reveal that mSTF is safe and feasible for gynaecologic cancer patients, however, this intervention requires high motivation. Moreover, mSTF during chemotherapy has the potential to reduce chemotherapy-induced toxicities and improve the tolerance of chemotherapy. Different blood parameters and metabolic parameters, particularly IGF-1 and insulin, were positively influenced by mSTF. A 6-day normocaloric KD prior to mSTF revealed no beneficial effects on either chemotherapy-induced toxicities or fasting-related discomfort and compliance. Clearly, there is a need for larger randomised controlled trials focussing on confirming the efficacy of mSTF accompanying chemotherapy as an innovative, supportive approach.

## Data Availability

The datasets generated and analysed during the study are available from the corresponding author on reasonable request.

## Abbreviations

ALP: Alkaline phosphatase
ALT: Alanine aminotransferase
AST: Aspartate aminotransferase
BIA: Bioelectrical impedance analysis
BMI: Body mass index
CRP: C-Reaktive Protein
CIPN: Chemotherapy-induced Polyneuropathy
EORTC: European Organisation for Research and Treatment of Cancer
FACIT: Functional Assessment of Chronic Illness Therapy
fT3: Free triiodothyronine
fT4: Free thyroxine
FU: Follow-up
GGT: Gamma-glutamyltransferase
IGF-1: Insulin-like growth factor-1
KD: Ketogenic diet
NC: Normocaloric diet
NCI CTCAE: National Cancer Institute Common Terminology Criteria for Adverse Events
mSTF: Modified short-term fasting
PE: Parameter estimate
QoL: Quality of life
T_0_: Baseline
TSH: Thyroid-stimulating hormone
SD: Standard errors
STF: Short-term fasting
